# Trait Emotional Intelligence Within Personality Space: Latent Profiles and Psychosocial Adjustment in Preadolescence

**DOI:** 10.3390/jintelligence14070136

**Published:** 2026-07-03

**Authors:** Constantinos M. Kokkinos, Ioanna Voulgaridou, Nafsika Antoniadou

**Affiliations:** 1 Department of Primary Education, Democritus University of Thrace, 68100 Alexandroupoli, Greece; 2 Department of Psychology, Democritus University of Thrace, 68300 Didymoteicho, Greece; ivoulg@psy.duth.gr; 3 Department of Primary Education, University of Ioannina, 45110 Ioannina, Greece; nantoniadou@uoi.gr; 4 School of Humanities, Hellenic Open University, 26335 Patras, Greece

**Keywords:** trait emotional intelligence, emotional intelligence, personality structure, individual differences, latent profile analysis, preadolescence, psychosocial adjustment, measurement modeling

## Abstract

Emotional intelligence and personality traits are key dimensions of individual differences contributing to developmental adjustment. This cross-sectional study investigated how trait emotional intelligence (trait EI), conceptualized as self-perceived dispositions closely embedded within personality hierarchies rather than a cognitive ability, is configured alongside the Big Five traits to influence socio-emotional functioning among preadolescents. Using a convenience sampling strategy, data were collected from 194 Greek preadolescents (aged 10–12) who completed self-report measures of trait EI, personality, and psychosocial adjustment via the Strengths and Difficulties Questionnaire (SDQ). Latent Profile Analysis (LPA) identified three distinct individual configurations: Emotionally Resourceful (54.6%), Emotionally Vulnerable (14.9%), and Emotionally Resilient (30.4%). Distal outcome testing via the Bolck-Croon-Hagenaars (BCH) three-step approach revealed that the Emotionally Resilient profile exhibited the most favorable adjustment (higher prosocial behavior, lower emotional/behavioral difficulties), whereas the Emotionally Vulnerable group displayed the highest psychosocial risks. While the reliance on cross-sectional self-reports requires cautious interpretation regarding causality, the findings underscore that preadolescent adjustment is better understood through integrated dispositional configurations rather than isolated traits, clarifying the role of trait EI within the broader personality space.

## 1. Introduction

Understanding how emotional competencies are structured within broader personality systems and how they jointly shape psychosocial adjustment is particularly important during preadolescence, a developmental period marked by increasing emotional complexity and growing reliance on peer relationships. Trait emotional intelligence (trait EI) refers to emotion-related self-perceptions and dispositions embedded within personality hierarchies and typically measured via self-report, distinct from ability EI (maximal performance) and many mixed models (Petrides, 2010; Petrides & Furnham, 2001). Given its conceptualization as part of the personality domain, a key issue concerns how trait EI relates to broader trait structures and whether it contributes meaningfully to understanding individual differences in psychosocial adjustment (De Raad, 2005; Petrides et al., 2007a; van der Linden et al., 2017). Recent systematic reviews further underscore the relevance of trait EI during developmental periods, showing consistent associations with psychological well-being, social functioning, and emotional adjustment in children and adolescents (Mancini et al., 2024; Özal et al., 2024). These findings highlight the importance of examining trait EI not in isolation, but within the broader constellation of personality traits that shape everyday emotional functioning.

Despite the extensive evidence linking both trait EI and broad personality traits to psychosocial adjustment, most existing research has relied on variable-centered approaches that examine associations at the population level. Such approaches, while informative, do not capture how emotional self-perceptions and personality traits co-occur within individuals. This represents a key gap in the literature, particularly during preadolescence, a period characterized by increasing heterogeneity in emotional and social functioning. Identifying latent configurations of trait EI and personality traits may therefore provide a more nuanced representation of individual differences, while also clarifying whether trait EI meaningfully differentiates profiles beyond general personality functioning.

Building on evidence that broad personality traits predict risk for psychopathology and adjustment outcomes (Kotov et al., 2010), the present study examines how trait EI considered jointly with the Big Five personality traits is associated with psychosocial adjustment (Andrei et al., 2016; Davis & Humphrey, 2012; Petrides et al., 2007a) using the Strengths and Difficulties Questionnaire (SDQ; Goodman, 1997). Utilizing a person-centered framework, we identify latent profiles and evaluate their associations with distal outcomes using robust three-step procedures that account for classification uncertainty (Asparouhov & Muthén, 2014). This integrated approach captures individual heterogeneity that standard population averages might otherwise obscure (Huttunen et al., 2025; Jiang et al., 2023).

### 1.1. Conceptualizing Trait Emotional Intelligence

A persistent challenge in the EI literature is that the term has been used to denote conceptually distinct constructs. A theoretically rigorous framework therefore begins by differentiating ability EI, trait EI, and mixed EI models, because each implies different mechanisms, measurement strategies, and validity claims. Ability EI conceptualizes EI as a set of cognitive-emotional abilities (e.g., perceiving emotions, understanding emotional meanings, using emotions to facilitate thought, and managing emotions) assessed via maximum-performance tests and treated as a form of intelligence aligned with cognitive capacities (Mayer et al., 2008). In this view, EI is closely aligned with traditional cognitive abilities and is expected to relate to performance-based outcomes. Mixed models often combine heterogeneous elements (e.g., traits, social competencies, motivational tendencies), which can broaden the construct at the expense of theoretical clarity and discriminant validity (Mayer et al., 2008; Petrides, 2010). By contrast, trait EI conceptualizes individual differences as self-perceived dispositions and habitual tendencies in emotion perception, expression, and regulation, best captured through self-report measures (Petrides, 2010; Petrides & Furnham, 2001) reflecting “typical performance”.

This distinction has important implications for construct validity. For trait EI, evidence must demonstrate coherent embedding in trait space, reliable measurement, and meaningful associations with outcomes where emotion-related dispositions are theoretically relevant (Petrides, 2010; Petrides et al., 2007a). Recent reviews further emphasize that trait EI shows consistent psychometric adequacy and developmental relevance across child and adolescent samples, supporting its use as a dispositional construct in younger populations (Özal et al., 2024).

Rather than treating EI as an intelligence in the same psychometric sense as maximum-performance ability models (Mayer et al., 2008), the current work examines how emotion-self-perceptions are organized within broader personality space during preadolescence (Petrides, 2010; Petrides & Furnham, 2001). In doing so, it contributes to the ongoing developmental literature by moving away from traditional variable-centered debates on incremental validity. Instead, it utilizes a person-centered framework to model how configurations of trait EI and Big Five traits naturally co-occur within individuals, examining how these integrated profiles are associated with psychosocial adjustment.

### 1.2. Trait EI Within Personality Structure and Overlap with the Big Five

Because trait EI belongs to the same ontological domain as personality traits (Petrides, 2010; Petrides & Furnham, 2001), it is expected to covary systematically with broader dispositional dimensions rather than functioning as an idiosyncratic aggregation of unrelated facets. Empirical work supports this view, showing that trait EI is meaningfully related to major personality dimensions while retaining a distinct configuration centered on emotion-related functioning (Petrides et al., 2007b).

Specifically, a substantial body of research indicates that trait EI is systematically embedded within the Big Five personality framework, with particularly strong and consistent associations with Neuroticism (negative), Extraversion, Agreeableness, and Conscientiousness (positive). Individuals high in trait EI tend to report lower emotional instability and higher sociability, empathy, and self-regulation (Petrides et al., 2007b; Pérez-González & Sánchez-Ruiz, 2014; Veselka et al., 2010). Moreover, structural models suggest that trait EI aligns with higher-order personality factors such as Stability and Plasticity (van der Linden et al., 2017), while genetic evidence indicates substantial shared variance with the Big Five traits (Veselka et al., 2010).

Although it has been argued that the behavioral content attributed to EI can largely be represented within existing trait frameworks (De Raad, 2005), trait EI theory interprets this overlap as theoretically expected rather than problematic (Petrides, 2010; Petrides & Furnham, 2001), suggesting that trait EI operates as a coherent dispositional system that provides a focused lens on emotional and social functioning (Mancini et al., 2024). Overall, the present study does not seek to establish the variable-centered incremental validity of trait EI over personality traits. Instead, the focus is on whether trait EI serves as a key differentiating feature when identifying integrated, holistic profiles of youth within that shared trait space.

### 1.3. Trait EI and Psychosocial Adjustment in Adolescence

Given its integration within personality structure, trait EI captures emotion-related aspects of broader dispositional functioning, reflecting a general tendency toward effective emotional adaptation (van der Linden et al., 2017).

This issue is particularly salient during preadolescence (approximately ages 10–12), a developmental transition marked by changes in emotional reactivity, increasing emotional complexity, growing self-awareness, and a heightened reliance on peer relationships (Somerville et al., 2013; Steinberg & Morris, 2001; Zimmermann & Iwanski, 2014). These shifts make individual differences in emotional self-perceptions especially relevant for daily functioning.

Consistent with this view, higher levels of trait EI have been associated with greater well-being, more adaptive coping, and fewer internalizing, externalizing, and social difficulties across youth populations (Andrei et al., 2016; Davis & Humphrey, 2012; Mancini et al., 2024; Petrides et al., 2007b). At the same time, broad personality traits, particularly neuroticism and conscientiousness, are well-established predictors of psychopathology and maladjustment (Kotov et al., 2010). These findings highlight the importance of examining trait EI within the broader personality framework, as its associations with psychosocial adjustment may reflect both shared variance with core traits and emotion-specific aspects of functioning (Petrides et al., 2007b; Siegling et al., 2015; Vernon et al., 2008). Moving beyond examining these components in isolation allows us to understand how they interactively co-occur within individuals.

### 1.4. A Person-Centered Approach to Personality and Emotional Functioning

While variable-centered approaches assume population homogeneity (Laursen & Hoff, 2006), this assumption may be overly restrictive in developmental contexts characterized by heterogeneity in psychological functioning (Bergman & Magnusson, 1997; Morin et al., 2018).

A person-centered approach, such as latent profile analysis (LPA), allows for the identification of subgroups defined by distinct configurations of trait EI and personality traits (Muthén & Muthén, 2000). This is conceptually consistent with the embedding of trait EI within personality space: individuals may cluster into profiles reflecting unique combinations of emotional self-perceptions and broader traits (Petrides et al., 2007b; Pérez-González & Sánchez-Ruiz, 2014). Recent studies adopting person-centered approaches in youth populations have demonstrated that distinct socio-emotional and behavioral profiles are meaningfully associated with academic, emotional, and social outcomes, supporting the utility of this approach for capturing developmental heterogeneity (Huttunen et al., 2025; Jiang et al., 2023).

Importantly, these profiles can highlight different patterns of psychosocial adjustment that traditional variable-centered methods might obscure (Laursen & Hoff, 2006; Morin et al., 2018). A key methodological consideration in this context is classification uncertainty. Because profiles are probabilistic, three-step approaches, such as the Bolck-Croon-Hagenaars (BCH) method, are utilized to examine differences in distal outcomes while preserving the latent structure, thereby providing robust, unbiased estimates (Asparouhov & Muthén, 2014).

### 1.5. The Present Study

Building on these considerations, the present study adopts a person-centered approach to examine whether distinct configurations of trait EI and the Big Five personality traits emerge in preadolescence, and whether these configurations map onto meaningful differences in psychosocial adjustment. Based on existing theory and empirical findings, it is expected that profiles reflecting more adaptive configurations (e.g., high trait EI, low neuroticism, and higher levels of conscientiousness and agreeableness) will be associated with more favorable psychosocial adjustment, whereas profiles characterized by lower trait EI and higher neuroticism will be associated with greater psychosocial difficulties.

In addition, a more moderate or balanced profile may emerge, reflecting intermediate levels across emotional intelligence and personality traits, and corresponding to average levels of psychosocial functioning. Differences in psychosocial adjustment across profiles are examined using the SDQ, with age and gender controlled for in the analyses. To ensure robust estimation of profile differences, distal associations are tested using three-step procedures that account for classification uncertainty (Asparouhov & Muthén, 2014). In doing so, the study aims to clarify whether trait EI contributes to the differentiation of latent personality profiles beyond general dispositional tendencies, thereby addressing a key unresolved issue in the literature.

## 2. Materials and Methods

### 2.1. Participants

The sample consisted of 194 preadolescents recruited from public upper elementary schools in urban areas of Greece. Participants’ ages ranged from 10 to 12 years (M = 11.32, SD = 0.72). Regarding gender, 81 participants were boys (41.8%) and 113 were girls (58.2%). All participants were enrolled in the two final grades of elementary education at the time of data collection, with 79 students (40.7%) attending the fifth grade and 115 students (59.3%) attending the sixth grade.

Participants were recruited using a convenience sampling strategy from multiple classrooms across several public schools. Of the 260 individuals who initially participated in the study, 194 provided complete and usable data and were retained for the final analyses, corresponding to a retention rate of 74.6%. The remaining 66 participants were excluded due to incomplete responses or invalid data, resulting in an attrition/exclusion rate of 25.4%. Because demographic information was not available for all excluded participants, comparisons between included and excluded cases could not be conducted.

The final sample size was considered adequate for the purposes of latent profile analysis, particularly given the relatively small number of indicators used and the clear separation observed among the identified profiles. Nevertheless, latent profile solutions based on modest sample sizes and profiles containing relatively few participants should be interpreted with caution, as smaller classes may be less stable and more sample-dependent.

Students were nested within classrooms; however, due to the limited number of participants per class, clustering effects were not modeled in the analyses. Furthermore, because participants were recruited through convenience sampling from public schools located in urban areas, the findings may not generalize to all Greek preadolescents, particularly those from rural regions or different educational and socioeconomic contexts.

### 2.2. Measures

#### 2.2.1. Demographic Information

Demographic characteristics were assessed using a brief self-report questionnaire that included items on participants’ gender, age, and grade level.

#### 2.2.2. Trait Emotional Intelligence

Trait emotional intelligence was assessed using the Trait Emotional Intelligence Questionnaire-Adolescent Short Form (TEIQue-ASF; Petrides et al., 2007a). The TEIQue-ASF is a simplified version of the adult short form, adapted linguistically and syntactically for adolescent populations. The questionnaire consists of 30 items, with two items representing each of the 15 facets of trait emotional intelligence. These facets include adaptability, assertiveness, emotion perception, emotion expression, emotion management, emotion regulation, impulsivity, interpersonal relationships, self-esteem, self-motivation, social awareness, stress management, empathy, happiness, and optimism. Sample items include: “I find it easy to talk about my feelings with other people”, “I can put myself in someone else’s position and understand how they feel”, and “I can deal effectively with stress.” Responses were provided on a 7-point Likert-type scale ranging from 1 = strongly disagree to 7 = strongly agree. Several items were reverse-scored prior to computing scale scores. In line with the recommendations of the scale developers, only the total trait emotional intelligence score was used in the present study, as internal consistency estimates for the individual facets were low. The total scale demonstrated acceptable internal consistency in the present sample (Cronbach’s α = 0.70).

The TEIQue-ASF has been widely used in adolescent populations and has demonstrated acceptable psychometric properties in youth samples, including studies conducted in Greek contexts (Kokkinos & Vlavianou, 2021; Stamatopoulou et al., 2018). Although the lower end of the age range in the present study (10 years) is slightly below the age range for which the instrument was originally developed, previous research has shown that children in late childhood can provide meaningful self-reports of emotional self-perceptions when age-appropriate questionnaire formats are used. The questionnaire was administered in a classroom setting, and participants were given standardized instructions and the opportunity to request clarification if they had trouble understanding any item. Nevertheless, the inclusion of younger participants should be considered when interpreting the findings, and future research may benefit from further examining the suitability of the measure in preadolescent samples.

#### 2.2.3. Personality

Personality traits were assessed using the Greek Big Five Questionnaire for Children-Short Form (GBFQ-C-SF; Markos & Kokkinos, 2017), which is based on the original Big Five Questionnaire for Children (BFQ-C; Barbaranelli et al., 2003). The short form consists of 30 items, with six items measuring each of the five personality dimensions: Extraversion, Agreeableness, Conscientiousness, Openness to Experience, and Emotional Instability (Neuroticism). Items were rated on a 5-point Likert scale ranging from 1 = almost never to 5 = almost always. In the present sample, internal consistency coefficients were satisfactory: Emotional Instability (α = 0.71), Extraversion (α = 0.69), Agreeableness (α = 0.74), Conscientiousness (α = 0.73), and Openness to Experience (α = 0.70).

#### 2.2.4. Psychosocial Adjustment

Psychosocial adjustment was measured using the Strengths and Difficulties Questionnaire (SDQ; Goodman, 1997), adapted for Greek adolescents. The SDQ is a widely used screening instrument designed to assess both difficulties and strengths in children and adolescents aged 11–16 years. The questionnaire consists of 25 items grouped into five subscales: Conduct Problems, Emotional Symptoms, Hyperactivity/Inattention, Peer Problems, and Prosocial Behavior. Items are rated on a 3-point scale (0 = not true, 1 = somewhat true, 2 = certainly true), with reference to behaviors and feelings experienced during the past six months. In the present study, internal consistency estimates ranged from low to moderate—Emotional Symptoms (α = 0.56), Conduct Problems (α = 0.52), Hyperactivity/Inattention (α = 0.55), Peer Problems (α = 0.61), and Prosocial Behavior (α = 0.67)—which is consistent with previous findings in Greek non-clinical samples, where SDQ subscales—particularly those assessing specific domains—often demonstrate modest reliability (e.g., Kokkinos et al., 2010; Giannakopoulos et al., 2009). This pattern has been noted in the broader SDQ literature and is partly attributable to the small number of items per subscale and the heterogeneity of behaviors assessed. Thus, the SDQ is more appropriate for broad screening than for detailed subscale interpretation. These considerations are considered in the interpretation of the findings.

### 2.3. Procedure

The study was conducted in accordance with ethical guidelines for research involving human participants. Ethical approval and authorization to conduct the study were granted by the first author’s Institutional Review Board, followed by the relevant educational authorities and the participating school principals. Classroom teachers were informed about the aims and procedures of the study, and suitable dates and times were scheduled to minimize disruption to the school program. Written parental consent was secured prior to participation, and students provided verbal assent before completing the questionnaires. Participation was voluntary, and students were informed that they could withdraw at any time without any academic consequences. Data collection took place during regular school hours in the students’ classrooms and lasted approximately one teaching hour. Questionnaires were administered collectively, with the presence of both the researcher and classroom teachers, who assisted in maintaining students’ concentration and orderly conduct. Prior to administration, students were informed about the purpose of the study and reassured that there were no right or wrong answers. Additional explanations were provided when necessary to clarify unfamiliar terms or statements. All questionnaires were distributed and collected exclusively by the researcher to ensure consistency and confidentiality. All responses were collected anonymously, and no identifying information was recorded. Data were stored securely and used exclusively for research purposes.

### 2.4. Data Analysis

Data analyses were conducted using IBM SPSS Statistics version 26 (IBM Corp., Armonk, NY, USA) and Mplus version 8.6 (Muthén & Muthén, 2017 Los Angeles, CA, USA). Descriptive statistics and Pearson correlation coefficients were computed for all study variables. Latent Profile Analysis was employed to identify distinct profiles based on Trait Emotional Intelligence and the Big Five personality traits (Neuroticism, Extraversion, Agreeableness, Conscientiousness, and Openness). All continuous indicators were standardized prior to the LPA. The LPA framework was used not merely as a classificatory tool, but as a person-centered modeling strategy for examining how trait EI is organized in relation to broader personality dimensions during preadolescence.

Models were estimated using robust maximum likelihood (MLR) estimation to account for potential deviations from normality. To avoid local maxima, a large number of random start values were specified (e.g., 500 initial stage starts and 100 final stage optimizations). Participants with incomplete or invalid data were excluded prior to the analyses, and all analyses were conducted using the final sample of 194 participants.

Model selection was guided by multiple statistical criteria, including the Akaike Information Criterion (AIC), Bayesian Information Criterion (BIC), sample-size adjusted BIC, entropy, and the Lo–Mendell–Rubin adjusted likelihood ratio test (LMR-LRT). Lower information criterion values and significant LMR-LRT results indicate superior model fit, while entropy values closer to 1.00 reflect better classification accuracy. In addition, model interpretability and parsimony were considered when determining the optimal number of profiles.

Associations between latent profile membership and psychosocial adjustment were primarily examined using the BCH three-step approach (Asparouhov & Muthén, 2014) implemented in Mplus version 8.6, which accounts for classification uncertainty and allows for unbiased estimation of distal outcome differences while preserving the latent profile structure.

For comparison purposes, a multivariate analysis of covariance (MANCOVA) was also conducted, with the five SDQ dimensions entered simultaneously as dependent variables and age and gender included as covariates to control for potential demographic effects. Follow-up univariate tests were used to interpret significant multivariate effects. These analyses are reported as supplementary and should be interpreted with caution, given that classification-based approaches may introduce bias in distal outcome comparisons.

Given the modest sample size and the relatively small size of one of the identified profiles, the results should be interpreted with caution, particularly with respect to the stability and generalizability of the latent profile solution.

## 3. Results

### 3.1. Preliminary Analysis

Means, standard deviations, and Pearson correlations among TEI, personality traits, and SDQ dimensions are presented in Table 1. Overall, higher emotional intelligence was associated with lower Neuroticism and fewer psychosocial difficulties, and with higher levels of adaptive personality traits and prosocial behavior. Neuroticism showed strong positive associations with emotional and behavioral difficulties and negative associations with adaptive traits.

### 3.2. Latent Profile Analysis

Latent Profile Analysis was conducted using TEI and the Big Five personality traits as continuous indicators. All indicators were standardized prior to analysis. Models with one to four profiles were estimated and compared. In all models, indicator means were freely estimated across profiles, whereas variances were constrained to be equal across profiles, and covariances among indicators were fixed to zero, reflecting a conditional independence model.

Model fit was evaluated using information criteria (AIC, BIC, and sample-size adjusted BIC), entropy, class sizes, the Lo–Mendell–Rubin adjusted likelihood ratio test (LMR-LRT), the Bootstrap Likelihood Ratio Test (BLRT), and theoretical interpretability. As shown in Table 2, information criterion values generally decreased as additional profiles were extracted. Although the four-profile solution yielded a slightly lower AIC value and a significant BLRT result (*p* = .041), it did not provide a significant improvement over the three-profile solution according to the LMR-LRT (*p* = .174). Furthermore, the BIC increased from the three-profile to the four-profile solution, and the smallest class decreased to 8.3% of the sample. By contrast, the three-profile solution demonstrated a significant improvement over the two-profile solution according to both the LMR-LRT (*p* = .023) and BLRT (*p* < .001), while retaining meaningful class sizes and theoretical interpretability. Therefore, the three-profile model was considered the most parsimonious and theoretically interpretable representation of the data.

The retained three-profile solution yielded acceptable, although not high, classification quality. Entropy was 0.75, suggesting moderate separation between profiles. Average posterior probabilities for the three profiles were 0.95, 0.86, and 0.92 for Profiles 1, 2, and 3, respectively, indicating moderate to acceptable classification accuracy. Nevertheless, profile membership should be interpreted probabilistically rather than reflecting perfectly distinct groups. Taken together, the entropy value, posterior probabilities, meaningful class sizes, and theoretical interpretability provided acceptable support for the retained three-profile solution.

**Figure 1:**
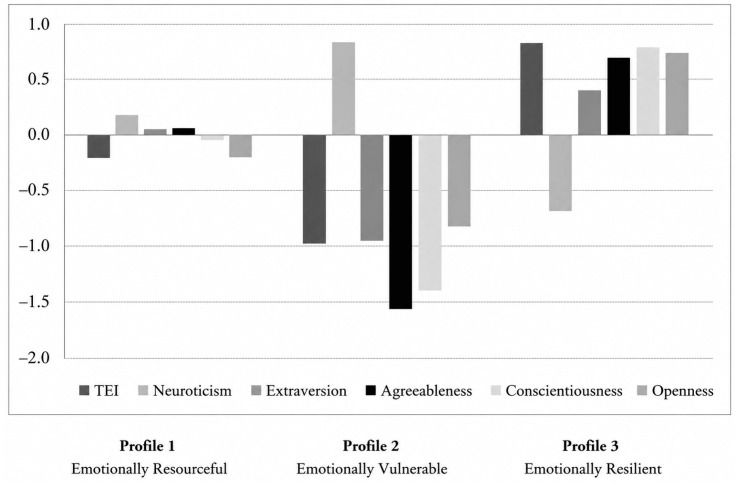
Standardized Mean Scores of Trait Emotional Intelligence and Big Five Personality Traits across the Three Latent Profiles.

The three latent profiles comprised 54.6% (*n* = 106), 14.9% (*n* = 29), and 30.4% (*n* = 59) of the sample, respectively. Figure 1 presents standardized mean scores of TEI and the Big Five personality traits across profiles. The first profile, labeled *Emotionally Resourceful*, was characterized by relatively high emotional intelligence, moderate levels of extraversion and openness, moderate-to-high conscientiousness and agreeableness, and moderate neuroticism. The second profile, labeled *Emotionally Vulnerable*, displayed lower emotional intelligence, high neuroticism, and comparatively lower levels of extraversion, agreeableness, and conscientiousness. The third profile, labeled *Emotionally Resilient*, was characterized by high emotional intelligence, high extraversion, agreeableness, conscientiousness, and openness, together with low neuroticism.

### 3.3. Psychosocial Adjustment Differences Across Profiles

To examine differences in psychosocial adjustment across the latent profiles while accounting for classification uncertainty, BCH three-step analyses were conducted for each SDQ dimension. These analyses were treated as the primary tests of profile differences because they preserve the latent profile structure and provide less biased estimates of distal outcome differences than classification-based approaches (Table 3).

Significant overall profile differences emerged across all five SDQ dimensions. Specifically, statistically significant profile differences were observed: prosocial behavior, χ^2^(2) = 73.73, *p* < .001; hyperactivity, χ^2^(2) = 76.01, *p* < .001; conduct problems, χ^2^(2) = 65.08, *p* < .001; peer problems, χ^2^(2) = 40.56, *p* < .001; and emotional symptoms, χ^2^(2) = 39.60, *p* < .001.

For prosocial behavior, Profile 3 (Emotionally Resilient) exhibited the highest levels (M = 2.85), followed by Profile 1 (Emotionally Resourceful; M = 2.46), whereas Profile 2 (Emotionally Vulnerable) showed the lowest levels (M = 2.15). All pairwise comparisons were statistically significant following Holm correction (*p*s < .01). Similarly, significant profile differences emerged for hyperactivity, conduct problems, and peer problems, with Profile 2 consistently displaying the highest levels of difficulties and Profile 3 the lowest. All pairwise comparisons reached statistical significance (*p*s ≤ .01). For emotional symptoms, the BCH analysis indicated significant overall profile differences, χ^2^(2) = 39.60, *p* < .001. Pairwise comparisons showed that Profiles 1 and 2 did not differ significantly from each other (*p* = .41), but both exhibited significantly higher levels of emotional symptoms than Profile 3 (*p*s < .001), yielding a pattern in which Profile 2 ≈ Profile 1 > Profile 3.

Overall, the BCH findings revealed a theoretically coherent pattern in which the Emotionally Resilient profile demonstrated the most favorable psychosocial adjustment, whereas the Emotionally Vulnerable profile displayed the highest levels of psychosocial difficulties.

As a supplementary analysis, a multivariate analysis of covariance (MANCOVA) was conducted to examine profile differences while statistically controlling for age and gender. Profile membership served as the independent variable, the five SDQ dimensions were entered as the multivariate dependent variables, and age and gender were included as covariates.

Preliminary assumption testing indicated a violation of the homogeneity of covariance matrices, Box’s M = 53.95, *p* = .009. In addition, Levene’s tests suggested violations of homogeneity of variances for prosocial behavior, conduct problems, and peer problems. Given these violations and the unequal group sizes, Pillai’s Trace was selected as the primary multivariate test statistic due to its robustness under such conditions.

At the multivariate level, there was a statistically significant effect of profile membership on psychosocial adjustment after controlling for age and gender (Pillai’s Trace = 0.514, F(10, 372) = 12.87, *p* < .001, partial η^2^ = 0.257), indicating a strong multivariate effect of latent profile membership on the overall pattern of psychosocial adjustment outcomes. Age did not exhibit a significant multivariate association with the SDQ dimensions (Pillai’s Trace = 0.014, *p* = .761), whereas gender showed a statistically significant multivariate effect (Pillai’s Trace = 0.158, F(5, 185) = 6.94, *p* < .001, partial η^2^ = 0.158).

Follow-up univariate analyses revealed statistically significant profile differences across all five SDQ dimensions after Holm correction: prosocial behavior, F(2, 189) = 29.36, partial η^2^ = 0.237; hyperactivity, F(2, 189) = 33.61, partial η^2^ = 0.262; conduct problems, F(2, 189) = 23.38, partial η^2^ = 0.198; peer problems, F(2, 189) = 18.50, partial η^2^ = 0.164; and emotional symptoms, F(2, 189) = 18.77, partial η^2^ = 0.166. The pattern of covariate-adjusted results closely paralleled the BCH findings, providing additional support for the observed profile differences.

Examination of the estimated marginal means, adjusted for age and gender, revealed a pattern that closely paralleled the BCH findings. Specifically, the Emotionally Resilient profile was characterized by higher levels of prosocial behavior and lower levels of emotional and behavioral difficulties, whereas the Emotionally Vulnerable profile exhibited the highest levels of psychosocial difficulties. Thus, the covariate-adjusted MANCOVA results provided additional support for the pattern of profile differences identified by the BCH analyses.

## 4. Discussion

The present study examined whether distinct configurations of trait EI and Big Five personality traits can be identified in preadolescence and whether these profiles differ in psychosocial adjustment. Using a person-centered approach, the findings highlight how combinations of emotional self-perceptions and personality dispositions are associated with multiple domains of adjustment during a critical developmental period. Rather than reiterating population-level trait overlaps discussed in the previous literature, our results provide an empirical overview of how these traits interactively combine within individual preadolescents to shape distinct psychological profiles.

Three distinct latent profiles emerged in this sample of Greek pre-adolescents, which were meaningfully differentiated across multiple domains of psychosocial adjustment. Across both the covariate-adjusted MANCOVA and BCH analyses, profile membership was associated with a strong multivariate pattern of SDQ outcomes, indicating that person-centered configurations capture substantively important heterogeneity in developmental functioning. This is consistent with recent person-centered research showing that distinct socio-emotional profiles in youth are differentially associated with adjustment-relevant outcomes (Huttunen et al., 2025; Jiang et al., 2023).

The pattern of findings was largely coherent. The Emotionally Vulnerable profile (low trait EI, high neuroticism, lower extraversion/agreeableness/conscientiousness) displayed the highest difficulties and the lowest prosocial behavior, whereas the Emotionally Resilient profile (high trait EI, low neuroticism, high adaptive traits) showed the most favorable adjustment. The Emotionally Resourceful profile (high trait EI with more moderate trait levels, including moderate neuroticism) generally occupied an intermediate position, although for emotional symptoms, it did not differ significantly from the Emotionally Vulnerable profile.

This profile solution aligns with the literature positioning trait EI within established personality structures while retaining functional relevance. Within this framework, the Emotionally Resilient profile can be interpreted as a synergistic configuration in which high trait EI co-occurs with adaptive personality characteristics, including low neuroticism and high extraversion, agreeableness, conscientiousness, and openness. Our data demonstrates that when high emotional self-efficacy converges with these traits, it provides evidence that trait EI can be anchored within broader personality frameworks and may reflect a higher-order tendency toward adaptive functioning expressed through emotional self-efficacy (Pérez-González & Sánchez-Ruiz, 2014; van der Linden et al., 2017). More recent syntheses further support this interpretation, indicating that trait EI is consistently associated with better emotional adjustment, well-being, and social functioning across youth populations (Mancini et al., 2024). Accordingly, this profile captures the vital convergence of emotional self-efficacy with broader dispositional resources, such as reduced vulnerability to negative affect and enhanced self-regulation, which together link to more favorable psychosocial functioning in early adolescence (Petrides et al., 2007b).

The Emotionally Vulnerable profile is equally interpretable within this integrative framework. Our findings demonstrate that the empirical combination of low trait EI and high neuroticism, along with reduced levels of other adaptive traits, reflects heightened emotional reactivity alongside weaker regulatory and interpersonal resources. This specific risk configuration is consistent with theoretical expectations that emotional vulnerability and lower self-perceived emotional competence co-occur, increasing risk for both internalizing and externalizing problems. Moreover, this observed data pattern aligns with meta-analytic evidence linking broad traits, particularly neuroticism, to anxiety, depression, and other psychopathology-relevant outcomes (Kotov et al., 2010). Recent work further corroborates our profile’s distinct vulnerabilities, suggesting that lower socio-emotional competencies are systematically associated with higher behavioral and emotional difficulties in school-aged populations (Jiang et al., 2023). By capturing these overlapping deficits simultaneously, our person-centered approach clarifies the severe, compounding nature of developmental risk within this specific preadolescent subgroup.

Our data shows that the most pronounced and consistent differences emerged for prosocial behavior, which was highest in the Emotionally Resilient profile and lowest in the Emotionally Vulnerable profile. This finding may reflect how distinct configurations of traits manifest as visible behavioral differences in our sample and extends the broader literature associating higher trait EI in youth with more favorable socioemotional outcomes, including better interpersonal functioning. For example, trait EI has been linked to socioemotional competence and broader adjustment indicators in early adolescence (Frederickson et al., 2012), while research in adolescents indicates that trait EI and related social competencies jointly relate to fewer emotional and behavioral difficulties (Poulou, 2014). More recent reviews converge on the conclusion that trait EI is reliably associated with prosocial behavior and social competence across developmental stages (Mancini et al., 2024; Özal et al., 2024). Moving beyond isolated, population-level traits, the present person-centered findings indicate that configurations combining high trait EI with adaptive personality traits may be associated with prosocial behavior. Specifically, within this sample, the Emotionally Resilient profile may reflect a configuration of emotional self-efficacy, lower neuroticism, and stronger interpersonal dispositions that is linked to more favorable prosocial functioning (Siegling et al., 2017; Stamatopoulou et al., 2018).

A similar pattern emerged for behavioral difficulties. The Emotionally Vulnerable profile was associated with higher levels of conduct problems and hyperactivity/inattention, whereas the Emotionally Resilient profile was associated with lower levels. This finding is consistent with evidence that trait EI is negatively associated with behavioral difficulties in youth (Gugliandolo et al., 2015). One plausible interpretation is that low trait EI reflects reduced perceived capacity for emotion regulation and stress management, consistent with its conceptualization as emotion-related self-efficacy within personality (Petrides, 2010; Petrides & Furnham, 2001). Empirical evidence further indicates that individuals lower in trait EI are more likely to report maladaptive coping strategies and greater emotional distress (Mikolajczak et al., 2007; Petrides et al., 2007b). When combined with higher neuroticism and lower conscientiousness and agreeableness evident in our vulnerable subgroup, this specific combination of traits appears to create a compounding effect that may increase impulsive responding and reduce behavioral regulation in emotionally demanding contexts (Kotov et al., 2010; Malouff et al., 2005; Tackett, 2006). Although the cross-sectional, self-reported design of the present design strictly does not permit causal inference, the observed pattern provides clear, descriptive evidence consistent with the view that trait EI contributes to behavioral adjustment through self-regulatory and socioemotional processes.

For emotional symptoms, our primary BCH analysis revealed that the Emotionally Resilient profile reported the lowest levels. This coherence within our configuration is consistent with prior research showing that emotional self-efficacy indicators associate strongly with adolescent mental health (Davis & Humphrey, 2012) and with broad meta-analytic evidence highlighting the value of trait EI across psychological outcomes (Andrei et al., 2016). Notably, our BCH results indicated that the Emotionally Resourceful and Emotionally Vulnerable profiles did not differ significantly on emotional symptoms, despite the distinct differences in trait EI between these two groups. This finding is theoretically and empirically informative within our sample context. It suggests that when moderate-to-high levels of neuroticism are present, trait EI may not be sufficient to offset emotional symptomatology, at least as captured by the SDQ in this age group. This interpretation of our data is consistent with the robust role of neuroticism in predicting internalizing difficulties (Kotov et al., 2010), as well as with evidence that the strength of associations involving trait EI varies across outcomes and contexts (Andrei et al., 2016). Thus, trait EI may be more discriminating for behavioral and interpersonal domains than for internalizing symptoms during preadolescence.

Profile differences in peer problems were also significant. The Emotionally Vulnerable profile showed the highest levels of peer problems, the Emotionally Resourceful profile showed intermediate levels, and the Emotionally Resilient profile showed the lowest levels. This pattern is consistent with the broader BCH findings, according to which the Emotionally Resilient profile displayed the most favorable psychosocial adjustment, whereas the Emotionally Vulnerable profile showed the highest psychosocial risks. The result supports the interpretation that high trait EI combined with low neuroticism and adaptive Big Five traits is associated with more favorable peer-related functioning (Frederickson et al., 2012). One possible interpretation grounded in our profile is that the significantly higher extraversion and openness characterizing our Emotionally Resilient subgroup, although generally adaptive, may be associated with greater exposure to complex peer dynamics and potential interpersonal conflict. Indeed, higher extraversion has been linked to increased social engagement, which may create opportunities for both positive interactions and conflict (Rubin et al., 2006), while openness has been associated with exploratory social behavior and diverse peer involvement (Denissen & Penke, 2008). Developmental research further indicates that increased peer involvement during late childhood is accompanied by more frequent and complex peer conflicts (Laursen & Pursell, 2009). Conversely, the Emotionally Resourceful group’s moderate levels of extraversion and openness might represent a social buffer in this age group, resulting in fewer peer friction points. However, because peer relationship dynamics were not directly tracked in this study, this interpretation remains speculative and should be treated with caution until confirmed by empirical designs that explicitly capture social exposure metrics.

An important interpretive question concerns the extent to which the present findings reflect the organizational role of trait EI within broad personality space, given their well-documented overlap (van der Linden et al., 2017). The current design does not directly test incremental validity using variable-centered approaches; rather, our profile configurations demonstrate how trait EI operates interactively within configurations of personality traits. Within this framework, our findings suggest that trait EI is meaningfully associated with psychosocial adjustment as part of broader dispositional patterns. This interpretation is consistent with previous research indicating that trait EI is related to adjustment outcomes and may provide useful information in conjunction with personality traits (Petrides et al., 2007a; Andrei et al., 2016; Davis & Humphrey, 2012). The present results extend this literature by showing that instead of contributing unique population variance, trait EI serves as a primary clustering element to form identifiable profiles that differ in developmental risk and resilience, a pattern also supported by recent person-centered work in youth (Huttunen et al., 2025; Jiang et al., 2023).

At the same time, the findings are also consistent with the possibility that what is captured by high trait EI in combination with low neuroticism and high conscientiousness/agreeableness within our Emotionally Resilient profile is closely aligned with a broad general adaptiveness factor (Pérez-González & Sánchez-Ruiz, 2014; van der Linden et al., 2017). This reinforces a balanced interpretation of our results: trait EI appears practically useful for identifying vulnerable youth profiles and understanding patterns of adjustment at an individual level. However, because the present study does not include population-level incremental validity analyses, any interpretations of uniqueness should be grounded in explicit tests of incremental effects and careful consideration of higher-order personality overlap.

Overall, the present findings contribute to the study of EI in several ways. First, they support the view that trait EI is meaningfully embedded within broader individual-differences structures, particularly the Big Five personality framework. Second, the results demonstrate that during preadolescence, trait EI acts as a key differentiating element in the emergence of distinguishable person-centered profiles that differ systematically in psychosocial functioning. By combining LPA with BCH distal outcome modeling, the study illustrates how emotional intelligence can be examined through configurations that capture heterogeneity in developmental pathways.

Methodologically, the study benefits from the use of LPA combined with robust procedures for examining profile differences. The selection of the three-profile solution was supported by fit indices, interpretability, and acceptable classification quality, in line with recommendations for mixture modeling (Nylund et al., 2007; Tein et al., 2013). The inability to retain a four-profile solution likely reflects model overextraction or insufficient differentiation, which is common in samples of this size (Lubke & Muthén, 2005; Marsh et al., 2009; Wang & Wang, 2020). Importantly, the use of the BCH three-step approach strengthens confidence in the findings, as it accounts for classification uncertainty while preserving the latent profile structure (Asparouhov & Muthén, 2014). The convergence between BCH and MANCOVA results further supports the robustness of the observed profile differences.

From an applied perspective, the findings highlight the potential value of identifying subgroups of youth characterized by distinct emotional and personality configurations at a single point in time. Our descriptive evidence regarding the Emotionally Vulnerable profile suggests that tracking joint trait low-points can be highly relevant for understanding concurrent variation in children’s self-reported adjustment difficulties. Previous school-aged research supports this screening utility (Frederickson et al., 2012; Poulou, 2014). At the same time, the results indicate that even relatively balanced profiles like the Emotionally Resourceful group may show domain-specific vulnerabilities, underscoring the importance of nuanced and multidimensional assessment approaches. 

Several limitations should be acknowledged. First, the study relied exclusively on self-report measures for trait EI, personality, and psychosocial adjustment, raising concerns about shared method variance and perceptual biases (Podsakoff et al., 2003). This reliance raises a distinct vulnerability to shared method variance (SMV), meaning that the observed configurations and their associations with outcomes may be partially inflated by common-source bias and consistent individual response tendencies rather than purely reflecting underlying behavioral distinctions (Podsakoff et al., 2003). Future research would benefit from incorporating multi-informant data (e.g., parent, teacher, or peer reports) to strengthen construct validity, particularly for social outcomes. Second, several SDQ subscales demonstrated low-to-moderate internal consistency, a limitation that warrants caution when interpreting domain-specific findings. Low reliability may attenuate observed associations and reduce the precision with which specific dimensions of psychosocial adjustment are assessed. Although previous research has supported the use of the SDQ in Greek samples (Giannakopoulos et al., 2009; Goodman, 2001), the relatively low reliability of some subscales suggests that findings involving individual SDQ dimensions should be interpreted cautiously and replicated using alternative indicators of psychosocial adjustment, including broader internalizing and externalizing composites or total difficulties scores. Third, the cross-sectional design precludes causal inferences. Longitudinal research is needed to examine the stability of trait EI personality profiles and their prospective associations with changes in psychosocial adjustment, particularly across key developmental transitions (Maxwell & Cole, 2007). Relatedly, the stability and replicability of the identified latent profiles remain uncertain and should be examined in future studies using longitudinal and larger samples. Fourth, although students were nested within classrooms and schools, potential clustering effects were not modeled, which may have influenced the observed associations. Fifth, the present study did not directly test the incremental validity of trait EI beyond the Big Five using variable-centered approaches. Therefore, conclusions regarding its unique contribution should be interpreted with caution. Finally, the sample was recruited using a convenience sampling strategy and was restricted to Greek public upper elementary school students from urban areas. Consequently, the findings may not generalize to preadolescents from different geographical regions, educational settings, or cultural contexts. In addition, the smallest (vulnerable) profile included a relatively small number of participants, which may affect the stability of the latent profile solution. Replication in larger, more diverse, and preferably randomly selected samples is necessary to assess the stability, replicability, and generalizability of the identified profiles across contexts and populations.

## 5. Conclusions

The present study identified three distinct latent profiles characterized by different configurations of trait emotional intelligence and Big Five personality traits in a sample of Greek preadolescents. These profiles were associated with meaningful differences across multiple domains of psychosocial adjustment, including prosocial behavior, emotional symptoms, behavioral difficulties, and peer problems. Profiles characterized by higher trait EI and more adaptive personality characteristics were generally associated with more favorable psychosocial functioning, whereas profiles characterized by lower trait EI and elevated neuroticism were associated with greater psychosocial difficulties.

These findings contribute to the growing literature examining trait EI within broader personality frameworks and support the value of person-centered approaches for understanding individual differences during preadolescence. Rather than operating as isolated characteristics, emotional self-perceptions and personality traits appear to form distinct configurations that are differentially associated with psychosocial adjustment. Future longitudinal and multi-informant research is needed to examine the stability, developmental significance, and practical utility of these profiles across diverse populations and contexts.

## Figures and Tables

**Table 1:** Means (M), Standard Deviations (SD), and Pearson Correlations among Study Variables (N = 194).

Variable	M	SD	1	2	3	4	5	6	7	8	9	10
1. Trait Emotional Intelligence	4.74	0.65										
2. Neuroticism	2.53	0.79	−0.37 **									
3. Extraversion	3.89	0.64	0.40 **	−0.07								
4. Agreeableness	4.02	0.66	0.36 **	−0.50 **	0.32 **							
5. Conscientiousness	3.84	0.75	0.40 **	−0.35 **	0.21 **	0.62 **						
6. Openness	3.59	0.72	0.46 **	−0.21 **	0.28 **	0.31 **	0.49 **					
7. SDQ Prosocial Behavior	2.53	0.41	0.35 **	−0.34 **	0.32 **	0.51 **	0.38 **	0.27 **				
8. SDQ Hyperactivity	1.73	0.44	−0.35 **	0.32 **	−0.16 *	−0.44 **	−0.63 **	−0.41 **	−0.30 **			
9. SDQ Conduct Problems	1.55	0.35	−0.26 **	0.56 **	−0.17 *	−0.53 **	−0.36 **	−0.24 **	−0.39 **	0.34 **		
10. SDQ Peer Problems	1.45	0.42	−0.40 **	0.29 **	−0.33 **	−0.27 **	−0.22 **	−0.23 **	−0.31 **	0.19 **	0.36 **	
11. SDQ Emotional Symptoms	1.65	0.44	−0.47 **	0.43 **	−0.13	−0.20 **	−0.17 *	−0.34 **	−0.10	0.30 **	0.35 **	0.39 **

*Note*. * *p* < .05. ** *p* < .01. Higher SDQ scores indicate greater difficulties, except for Prosocial Behavior.

**Table 2:** Fit Indices for Latent Profile Models.

Number of Profiles	AIC	BIC	Sample-Adjusted BIC			Entropy	LMR-LRT *p*	BLRT *p*	Smallest Class (%)
1-profile	2491.44	2530.66	2492.64			—	—	—	—
2-profile	2289.90	2351.99	2291.80			0.79	<0.001	<0.001	33.5%
3-profile	2239.74	2324.71	2242.34	0.75	0.023	<0.001	14.9%		
4-profile	2228.89	2335.41	2232.21	0.78	0.174	0.041	8.3%		

*Note*. AIC = Akaike Information Criterion; BIC = Bayesian Information Criterion; LMR-LRT = Lo–Mendell–Rubin adjusted likelihood ratio test; BLRT = Bootstrap Likelihood Ratio Test. Significant LMR-LRT and BLRT values indicate that the k-profile solution fits significantly better than the k − 1 profile solution. The three-profile solution was retained because it demonstrated a lower BIC value than the four-profile solution, a significant improvement over the two-profile solution according to both the LMR-LRT and BLRT, acceptable (though not high) classification accuracy (entropy = 0.75), meaningful class sizes, and theoretical interpretability. Although the BLRT remained significant for the four-profile solution, the LMR-LRT was non-significant (*p* = .174), the BIC increased, and the smallest class contained only 8.3% of the sample.

**Table 3:** BCH Estimates of Psychosocial Adjustment (SDQ) Across Latent Profiles.

SDQ Dimension	Profile 1 Emotionally Resourceful (*n* = 106)	Profile 2 Emotionally Vulnerable (*n* = 29)	Profile 3 Emotionally Resilient (*n* = 59)	BCH χ^2^(2)	Pairwise Comparisons
Prosocial behavior	2.46 (0.07) [2.32, 2.60]	2.15 (0.10) [1.95, 2.35]	2.85 (0.06) [2.73, 2.97]	73.73 ***	P3 > P1 > P2
Hyperactivity	1.86 (0.06) [1.73, 2.02]	2.10 (0.08) [1.92, 2.30]	1.34 (0.10) [1.24, 1.45]	76.01 ***	P2 > P1 > P3
Conduct problems	1.62 (0.09) [1.51, 1.80]	1.85 (0.06) [1.72, 2.06]	1.30 (0.09) [1.19, 1.43]	65.08 ***	P2 > P1 > P3
Peer problems	1.51 (0.10) [1.38, 1.64]	1.79 (0.06) [1.62, 1.91]	1.18 (0.07) [1.05, 1.31]	40.56 ***	P2 > P1 > P3
Emotional symptoms	1.74 (0.08) [1.59, 1.91]	1.81 (0.08) [1.69, 2.02]	1.32 (0.08) [1.19, 1.43]	39.60 ***	P2 = P1 > P3

*Note.* Values represent BCH-estimated means with standard errors (SEs) and 95% confidence intervals. BCH = Bolck–Croon–Hagenaars three-step procedure. Pairwise comparisons were evaluated using Holm-adjusted *p* values. BCH estimates are unadjusted for age and gender but account for classification uncertainty. *** *p* < .001.

## Data Availability

Dataset available upon request from the authors.

## Abbreviations

The following abbreviations are used in this manuscript:

EI	Emotional Intelligence
trait EI	Trait Emotional Intelligence
ability EI	Ability Emotional Intelligence
LPA	Latent Profile Analysis
SDQ	Strengths and Difficulties Questionnaire
BCH	Bolck-Croon-Hagenaars three-step approach/BCH three-step approach
MANCOVA	Multivariate Analysis of Covariance
TEI	Trait Emotional Intelligence
TEIQue-ASF	Trait Emotional Intelligence Questionnaire-Adolescent Short Form
GBFQ-C-SF	Greek Big Five Questionnaire for Children, Short Form
BFQ-C	Big Five Questionnaire for Children
MLR	Robust Maximum Likelihood
FIML	Full Information Maximum Likelihood
AIC	Akaike Information Criterion
BIC	Bayesian Information Criterion
LMR-LRT	Lo-Mendell-Rubin adjusted likelihood ratio test
M	Mean
SD	Standard Deviation
N/n	Total sample size/subgroup size
p	Probability value/significance level
F	F statistic
χ^2^	Chi-square statistic
η^2^	Eta squared/effect size
α	Cronbach’s alpha
pp.	Pages

## References

[B1-jintelligence-14-00136] Andrei F., Siegling A. B., Aloe A. M., Baldaro B., Petrides K. V. (2016). The incremental validity of the Trait Emotional Intelligence Questionnaire (TEIQue): A systematic review and meta-analysis. Journal of Personality Assessment.

[B2-jintelligence-14-00136] Asparouhov T., Muthén B. (2014). Auxiliary variables in mixture modeling: Three-step approaches using Mplus. Structural Equation Modeling: A Multidisciplinary Journal.

[B3-jintelligence-14-00136] Barbaranelli C., Caprara G. V., Rabasca A., Pastorelli C. (2003). A questionnaire for measuring the Big Five in late childhood. Personality and Individual Differences.

[B4-jintelligence-14-00136] Bergman L. R., Magnusson D. (1997). A person-oriented approach in research on developmental psychopathology. Development and Psychopathology.

[B5-jintelligence-14-00136] Davis S. K., Humphrey N. (2012). Emotional intelligence predicts adolescent mental health beyond personality and cognitive ability. Personality and Individual Differences.

[B6-jintelligence-14-00136] Denissen J. J. A., Penke L. (2008). Motivational individual reaction norms underlying the Five-Factor model of personality: First steps toward a theory-based conceptual framework. Journal of Research in Personality.

[B7-jintelligence-14-00136] De Raad B. (2005). The trait-coverage of emotional intelligence. Personality and Individual Differences.

[B8-jintelligence-14-00136] Frederickson N., Petrides K. V., Simmonds E. (2012). Trait emotional intelligence as a predictor of socioemotional outcomes in early adolescence. Personality and Individual Differences.

[B9-jintelligence-14-00136] Giannakopoulos G., Tzavara C., Dimitrakaki C., Kolaitis G., Rotsika V., Tountas Y. (2009). The factor structure of the strengths and difficulties questionnaire (SDQ) in Greek adolescents. Annals of General Psychiatry.

[B10-jintelligence-14-00136] Goodman R. (1997). The strengths and difficulties questionnaire: A research note. Journal of Child Psychology and Psychiatry.

[B11-jintelligence-14-00136] Goodman R. (2001). Psychometric properties of the strengths and difficulties questionnaire. Journal of the American Academy of Child & Adolescent Psychiatry.

[B12-jintelligence-14-00136] Gugliandolo M. C., Costa S., Cuzzocrea F., Larcan R., Petrides K. V. (2015). Trait emotional intelligence and behavioral problems among adolescents: A cross-informant design. Personality and Individual Differences.

[B13-jintelligence-14-00136] Huttunen I., Upadyaya K., Salmela-Aro K. (2025). Adolescents’ social-emotional skills profiles, relationships at school, school anxiety, and educational aspirations. European Journal of Psychology of Education.

[B14-jintelligence-14-00136] Jiang N., Gao R., DiStefano C., Liu J., Weist M., Splett J. W., Halliday-Boykins C. A. (2023). Social-emotional and behavioral functioning profiles and demographic factors: A latent profile analysis in elementary students. Journal of Psychoeducational Assessment.

[B15-jintelligence-14-00136] Kokkinos C. M., Panayiotou G., Charalambous K., Antoniadou N., Davazoglou A. (2010). Greek EPQ-J: Further support for a three-factor model of personality in children and adolescents. Journal of Psychoeducational Assessment.

[B16-jintelligence-14-00136] Kokkinos C. M., Vlavianou E. (2021). The moderating role of emotional intelligence in the association between parenting practices and academic achievement among adolescents. Current Psychology.

[B17-jintelligence-14-00136] Kotov R., Gamez W., Schmidt F., Watson D. (2010). Linking “big” personality traits to anxiety, depressive, and substance use disorders: A meta-analysis. Psychological Bulletin.

[B18-jintelligence-14-00136] Laursen B., Hoff E. (2006). Person-centered and variable-centered approaches to longitudinal data. Merrill-Palmer Quarterly.

[B19-jintelligence-14-00136] Laursen B., Pursell G., Rubin K. H., Bukowski W. M., Laursen B. (2009). Conflict in peer relationships. Handbook of peer interactions, relationships, and groups.

[B20-jintelligence-14-00136] Lubke G. H., Muthén B. (2005). Investigating population heterogeneity with factor mixture models. Psychological Methods.

[B21-jintelligence-14-00136] Malouff J. M., Thorsteinsson E. B., Schutte N. S. (2005). The relationship between the five-factor model of personality and symptoms of clinical disorders: A meta-analysis. Journal of Psychopathology and Behavioral Assessment.

[B22-jintelligence-14-00136] Mancini G., Özal Z., Biolcati R., Trombini E., Petrides K. V. (2024). Trait emotional intelligence and adolescent psychological well-being: A systematic review. International Journal of Adolescence and Youth.

[B23-jintelligence-14-00136] Markos A., Kokkinos C. M. (2017). Development of a short form of the Greek Big Five Questionnaire for Children (GBFQ-C-SF): Validation among preadolescents. Personality and Individual Differences.

[B24-jintelligence-14-00136] Marsh H. W., Lüdtke O., Trautwein U., Morin A. J. S. (2009). Classical latent profile analysis of academic self-concept dimensions: Synergy of person- and variable-centered approaches. Structural Equation Modeling.

[B25-jintelligence-14-00136] Maxwell S. E., Cole D. A. (2007). Bias in cross-sectional analyses of longitudinal mediation. Psychological Methods.

[B26-jintelligence-14-00136] Mayer J. D., Salovey P., Caruso D. R. (2008). Emotional intelligence: New ability or eclectic traits?. American Psychologist.

[B27-jintelligence-14-00136] Mikolajczak M., Petrides K. V., Hurry J. (2007). Adolescents choosing self-harm as an emotion regulation strategy: The protective role of trait emotional intelligence. British Journal of Clinical Psychology.

[B28-jintelligence-14-00136] Morin A. J. S., Bujacz A., Gagné M. (2018). Person-centered methodologies in the organizational sciences: Introduction to the feature topic. Organizational Research Methods.

[B29-jintelligence-14-00136] Muthén B., Muthén L. (2000). Integrating person-centered and variable-centered analyses: Growth mixture modeling with latent trajectory classes. Alcoholism: Clinical and Experimental Research.

[B30-jintelligence-14-00136] Muthén B., Muthén L. (2017). Mplus. Handbook of item response theory.

[B31-jintelligence-14-00136] Nylund K. L., Asparouhov T., Muthén B. (2007). Deciding on the number of classes in latent class analysis and growth mixture modeling: A Monte Carlo simulation study. Structural Equation Modeling.

[B32-jintelligence-14-00136] Özal Z., Ambrosini F., Biolcati R., Trombini E., Petrides K. V. (2024). Exploring emotional intelligence in children using the Trait Emotional Intelligence Questionnaire: A systematic review. BMC Psychology.

[B33-jintelligence-14-00136] Petrides K. V. (2010). Trait emotional intelligence theory. Industrial and Organizational Psychology.

[B34-jintelligence-14-00136] Petrides K. V., Furnham A. (2001). Trait emotional intelligence: Psychometric investigation with reference to established trait taxonomies. European Journal of Personality.

[B35-jintelligence-14-00136] Petrides K. V., Pérez-González J. C., Furnham A. (2007a). On the criterion and incremental validity of trait emotional intelligence. Cognition & Emotion.

[B36-jintelligence-14-00136] Petrides K. V., Pita R., Kokkinaki F. (2007b). The location of trait emotional intelligence in personality factor space. British Journal of Psychology.

[B37-jintelligence-14-00136] Pérez-González J. C., Sánchez-Ruiz M. J. (2014). Trait emotional intelligence anchored within the Big Five, Big Two and Big One frameworks. Personality and Individual Differences.

[B38-jintelligence-14-00136] Podsakoff P. M., MacKenzie S. B., Lee J.-Y., Podsakoff N. P. (2003). Common method biases in behavioral research: A critical review. Journal of Applied Psychology.

[B39-jintelligence-14-00136] Poulou M. S. (2014). How are trait emotional intelligence and social skills related to emotional and behavioural difficulties in adolescents?. Educational Psychology.

[B40-jintelligence-14-00136] Rubin K. H., Bukowski W. M., Parker J. G., Damon W., Lerner R. M. (2006). Peer interactions, relationships, and groups. Handbook of child psychology.

[B41-jintelligence-14-00136] Siegling A. B., Furnham A., Petrides K. V. (2015). Trait emotional intelligence and personality: Gender-invariant linkages across different measures of the Big Five. Journal of Psychoeducational Assessment.

[B42-jintelligence-14-00136] Siegling A. B., Vesely A. K., Saklofske D. H., Frederickson N., Petrides K. V. (2017). Incremental validity of the Trait Emotional Intelligence Questionnaire–Adolescent Short Form (TEIQue–ASF). European Journal of Psychological Assessment.

[B43-jintelligence-14-00136] Somerville L. H., Jones R. M., Casey B. J. (2013). A time of change: Behavioral and neural correlates of adolescent sensitivity to social evaluation. Current Directions in Psychological Science.

[B44-jintelligence-14-00136] Stamatopoulou M., Galanis P., Tzavella F., Petrides K. V., Prezerakos P. (2018). Trait Emotional Intelligence Questionnaire-Adolescent Short Form: A psychometric investigation in Greek context. Journal of Psychoeducational Assessment.

[B45-jintelligence-14-00136] Steinberg L., Morris A. S. (2001). Adolescent development. Annual Review of Psychology.

[B46-jintelligence-14-00136] Tackett J. L. (2006). Evaluating models of the personality–psychopathology relationship in children and adolescents. Clinical Psychology Review.

[B47-jintelligence-14-00136] Tein J.-Y., Coxe S., Cham H. (2013). Statistical power to detect the correct number of classes in latent profile analysis. Structural Equation Modeling.

[B48-jintelligence-14-00136] van der Linden D., Pekaar K. A., Bakker A. B., Schermer J. A., Vernon P. A., Dunkel C. S., Petrides K. V. (2017). Overlap between the general factor of personality and emotional intelligence: A meta-analysis. Psychological Bulletin.

[B49-jintelligence-14-00136] Vernon P. A., Villani V. C., Schermer J. A., Petrides K. V. (2008). Phenotypic and genetic associations between the Big Five and trait emotional intelligence. Twin Research and Human Genetics.

[B50-jintelligence-14-00136] Veselka L., Schermer J. A., Petrides K. V., Vernon P. A. (2010). Evidence for a heritable general factor of personality in two studies. Twin Research and Human Genetics.

[B51-jintelligence-14-00136] Wang J., Wang X. (2020). Structural equation modeling: Applications using Mplus.

[B52-jintelligence-14-00136] Zimmermann P., Iwanski A. (2014). Emotion regulation from early adolescence to emerging adulthood. International Journal of Behavioral Development.

